# The boundary conditions of the liking bias in moral character judgments

**DOI:** 10.1038/s41598-022-22147-7

**Published:** 2022-10-14

**Authors:** Konrad Bocian, Katarzyna Myslinska Szarek, Katarzyna Miazek, Wieslaw Baryla, Bogdan Wojciszke

**Affiliations:** Department of Psychology in Sopot, SWPS University of Social Sciences and Humanities, Polna 16/20, 81-745 Sopot, Poland

**Keywords:** Human behaviour, Psychology

## Abstract

Recent research has shown that moral character judgments are prone to the liking bias—well-liked people are seen as morally superior to disliked or neutral ones. However, whether moral information about their past behavior would moderate the liking bias is still an open empirical question addressed in present studies. In Study 1 (*N* = 653), participants updated their biased moral character impressions when moral information about the target was introduced after the liking induction. In preregistered Study 2 (*N* = 601), when moral information about the target was presented before the liking induction, moral information had a stronger impact on moral character judgments than liking. Study 3 (*N* = 398) showed that moral character impression updating was three times greater when moral information was presented after (vs. before) the attitude induction. Further analyses of changes in participants’ moral judgments certainty revealed that moral information reduced their uncertainty stronger than attitudes. In effect, the latter were more amenable to updating than information-based judgments. Thus, we present evidence that moral information updates moral character impressions biased by liking. Nevertheless, liking also, but to a lesser extent, updates moral character impressions initially grounded on moral information. We propose that certainty about others’ moral character explains when and how moral information limits the impact of attitudinal influences on moral character judgments.

## Introduction

Sometimes people whom we like behave unethically. For example, in 2004, Martha Stewart was sentenced to 5 months for obstruction of justice and lying to federal investigators^1^. As research on the liking bias suggests that interpersonal attitudes have a profound impact on morality judgments^2–5^, one could argue that a positive attitude toward Martha would reduce the adverse effects of her immoral behavior, so she will still be perceived as a moral person. However, numerous studies show that moral information is critical in determining the overall impression of individuals and groups^6^. Especially immoral actions carry more weight towards updating the impression than moral actions—the effect commonly known as the *negativity bias* in impression formation^7,8^. This suggests that Martha’s immoral behavior would impact people’s inferences of her moral character more than a positive attitude toward her. Consequently, she would be perceived as immoral.

This paper investigates to what extent moral information constrains the impact of the liking bias on moral character attributions—liked people are seen as more moral than disliked or neutral ones. Specifically, we aimed to test whether moral information updates moral character impressions trigged by liking. We also tested if liking corrects the moral character impressions triggered by moral information. Based on Bayesian inference models^9^, we argue that liking produces a weak prior belief about others’ moral character. This belief should be updated in the face of new information about others’ moral behavior. Conversely, as the information about others’ moral behavior produces a solid prior belief about their’ moral character, liking should not update this belief.

## Liking bias in moral character judgments

The idea of liking bias in moral judgments is based on theoretical assumptions that an egocentric perspective shapes every social judgment, including morality^10–12^. Evidence from past studies confirms that the egocentric perspective contributes to many errors in social^13–15^ and justice judgments^16–18^. In the same vein, studies have shown that the egocentric perspective shapes judgments regarding not only the moral behavior of individuals^19,20^ and in-group members^21,22^ but also rules^23^ and statutes^24^. People make these errors because egocentrism is automatic—people experience the world directly, which is fast and effortless, while taking a perspective of others requires effort and cognitive resources^11,12^.

Indisputably, interpersonal attitudes constitute a substantial facet of the egocentric perspective, so they should strongly influence moral judgments, probably quickly and automatically. For example, there is evidence that moral traits increase liking when morality is advantageous for a perceiver’s goals, but this preference is eliminated when immorality is goal-conducive^5^. Moreover, research has shown that even orthogonal manipulation of judgments of a character’s morality and likability cannot suppress their relationship^4^. Finally, morality is the most critical factor in liking, respecting, and knowing a person^25^, which confirms the assumption of the affective disposition theory^26^, which argues that people judge others as moral because they like them.

As the egocentric perspective is fast and automatic, there is a high probability that moral character judgments could be biased by liking. Recent research has directly tested whether liking (vs. disliking) distorts moral judgments. Specifically, it was found that three different liking induction methods not related to morality (belief similarity, mimicking, and mere exposure) influenced moral character judgments, so a well-liked person was judged as more moral than a disliked one, the effect called the liking bias^3,27^.

This evidence corresponds with studies that showed that people infer moral character quickly and without effort^6,28,29^. For example, after exposure to novel faces, people need as little as 100 ms to infer stable judgments about others’ trustworthiness^30^. Further studies confirmed that these impressions are made even when facial information is not reached by conscious awareness^31^. Therefore, researchers agree that major sources of biases in intuitive judgments, including judgments of moral character, are automatic^32^. As the egocentric perspective is intuitive and inextricably linked to interpersonal attitudes, liking is a vital source of bias in moral character judgments.

In the present research, we attempt to combine recent evidence on the liking bias and impression updating (discussed below) to investigate the interplay between attitudes and moral information in moral character inferences. Specifically, we examine whether the moment of the moral information presentation (before or after liking induction) would moderate the liking bias in moral character judgments. Research on impression updating indicates that the liking bias would be attenuated or eliminated after moral information presentation. However, research on the liking bias suggests that after moral information presentation, liking would still bias moral character attributions.

## Uncertainty, moral inferences, and impression updating

There is an abundance of evidence that moral information dominates impression development. For example, global impressions of others are more influenced by moral traits than traits related to competence^33^ or warmth^28^. Moreover, when people gather information about others, they are more interested in obtaining their morality than competence^33^ or sociability^34^. Finally, adding moral information substantially impacts impression changes more than adding information on sociability or competence^35^. In the light of robust evidence confirming the primacy of morality in impression formation, researchers proposed a new framework of person and group perception: The Moral Primacy Model (MPM) of impression development. According to the MPM, moral information dominates each stage of impression formation: gathering information, first impressions, and revising the impression^6^. Therefore, we argue that moral information should moderate the impact of liking bias on moral character judgments.

Impression formation is a dynamic process. Numerous studies have demonstrated that people update their impression of others in the light of incoming new information, even if it is inconsistent with prior knowledge^36,37^. However, not each piece of information carries the same weight in the impression updating process^38^. A classic study has shown that impression formation is prone to negativity bias because immoral information impacts impression updating more than moral information^39^. Further research has found evidence that negative behaviors related to morality, in contrast to positive behaviors, are perceived as more diagnostic. This could explain why people consider immoral information (vs. moral) more important in the impression updating process^8^.

Although research on the negativity bias suggests that people are less willing to update their negative than positive character impressions^7,8^, recent research has proven that beliefs about the morality of bad agents are more uncertain than beliefs about the morality of good agents and, therefore more amenable to updating^40^. This result corresponds with evidence that threatening stimuli are arousing^41^ when arousal increases belief uncertainty^42^, and uncertain (vs. certain) attitudes are more amenable to change^43^. Finally, uncertainty generates aversive reactions in both non-social^44^ and social domains^37^, so people are strongly motivated to reduce it^45^. How do people reduce uncertainty in a social world?

According to the model of social uncertainty^46^, people are intrinsically motivated to reduce uncertainty triggered by social stimuli and the attendant negative affect with three interrelated mechanisms: automatic inference, controlled inference, and social learning. An automatic deduction is activated without effort and is largely unaffected by other ongoing mental processes. In contrast, a controlled inference is a process that updates automatic first impressions in light of incoming information at the expense of increased effort and cognitive control^46^. Because automatic and controlled processes are best explained as forming a continuum rather than a dichotomy^47^, we may assume that social stimuli can trigger less or more automatic processes depending on the available information.

Similarly, based on Bayesian inference models, Crockett^9^ suggest that a weak prior belief about a target’s moral character is more malleable to updating in line with new evidence than a strong initial belief. Therefore, we argue that interpersonal liking or disliking starts mostly automatically and results in less certain inferences about others’ moral character (weak prior) than moral information, which triggers primarily controlled and more safe inference (strong prior). As a result, moral information updates moral character judgments biased by liking more strongly than liking updates moral character judgments based on moral information.

## Overview of the present studies

In the present studies, we had three goals. First, we aimed to replicate and extend prior work on the liking bias, which found that liking influences moral character judgments independently of how the liking was created^3,27^. In Study 1, participants’ facial expressions were mimicked or not by a target person, while in Study 2 (preregistered) and Study 3, we convinced participants that the target person had similar or dissimilar personal preferences to their own. We hypothesized that participants would like the target person more when their facial expressions would be mimicked (vs. not mimicked) or when the target person would display similar (vs. dissimilar) personal preferences as participants. We also expected more (vs. less) favorable moral character judgments for the target who mimicked (vs. not) the participant’s facial expressions or had the same (vs. different) personal preferences as participants.

Second, we investigated if presenting participants with information regarding the past behavior of the target person would limit the influence of liking on moral character judgments. Specifically, we manipulated whether the given behavior was moral, immoral, or neutral (Study 1 and 2) or only immoral (Study 3). We hypothesized that adding morally relevant information, especially negative, should lead participants to update their moral character impressions since moral information is more diagnostic and triggers more controlled inferences than interpersonal attitudes. However, we also assumed that liking would not affect moral character impression updating if the first impression is built on moral information. Therefore, in Study 3, we manipulated whether moral information was presented before or after the attitude induction. Moreover, we measured participants’ moral character judgments twice before and after the moral information and attitude, induction to investigate changes in moral character impression updating.

Finally, to investigate the underlying process of moral character impression updating, in Study 3, we measured the extent to which participants were certain about their moral character judgments after the moral information and attitude induction. We assumed that attitude’s similarity or dissimilarity produces a weak prior belief about others’ moral character. As a result, this belief should be updated with the second information about others’ immoral past. Oppositely, as the information about others’ immoral past should produce a solid prior conviction about their moral character, liking-disliking (induced by belief similarity or dissimilarity) should not update this belief.

This article reports all measures, all manipulations, and any data exclusions. Any additional measures not included in the primary analyses are described in the Supplement. The reported studies were approved by the ethical committee of the SWPS University (Ethics Clearance ID: WKE/S 2021/6/IV/101) and were performed in accordance with guidelines and regulations of the Institutional Ethics Committee at the Faculty of Psychology, SWPS University. All participants provided informed consent.

## Study 1

In Study 1, we induced (or not) a positive attitude toward a target person by mimicking (or not) the participants’ facial expressions by the target. Next, the participants were presented with information about the target’s past moral or immoral behavior in the workplace. We expected that mimicry would generate a higher liking toward the target, and as a result, participants would judge the target as more moral. In contrast, a lack of mimicry should generate less liking and lower judgments of moral character. However, we also predicted that this main effect would be moderated by the information about the target’s past moral behavior because moral information is more diagnostic and provides more certainty than interpersonal attitudes.

### Method

#### Participants and procedure

To estimate the desired sample size for Study 1, we used Giner-Sorolla’s recommendations for powering interactions^48^. According to Study 4 of Bocian et al.^3^, the correlation coefficient between morality and liking was *r* = 0.47. Using G*Power^49^, we estimated the target sample size to be *N* = 48 (assuming a power of 0.95, two-tailed) to replicate this effect. Because we expected a 50% attenuation in the moral information condition, we increased the sample size 14 times, which resulted in a target of 672 participants. Using the university pooling sample, we recruited 653 Polish participants (445 women; mean age = 23.97 years, *SD* = 5.89). Based on a sensitivity power analysis, this sample size provides 0.80 power to detect an effect size of *f*^2^ = 0.12.

We used a computer-based method for the attitude manipulation that involves mimicking participants’ facial expressions^50^. Participants were convinced that they would participate in a live interaction with another person (the target) via video chat. In reality, they interacted with the professional actress recorded before, and participants watched a movie clip synchronized with the prompts given to them via computer headphones. We asked participants to express different basic emotions (e.g., anger, surprise) to the person visible on the screen (the target), who would try to guess what emotion they expressed. Participants were randomly allocated in the mimicry condition or the no-mimicry condition. In the first condition, the target expressed emotions shown by the participants immediately after they expressed them. In the second condition, the target’s face was still and did not express any emotions.

Further, we told the participants that they would see a short employee assessment form written by the target’s supervisor (see the Supplement for a description of the forms). We asked participants to read the assessment carefully because later, they would be asked to answer some questions about the target. We randomly presented to participants the assessment form in which the target’s supervisor mentioned the target’s moral behavior in the workplace (the moral condition), immoral behavior (the immoral condition), or information in which the morality was omitted (the control condition). All conditions were similar in length and conveyed the same information. The only difference regarded the target’s behavior. All three conditions were pretested in a pilot study (see the Supplement for the pilot study results). For example, in the control condition, participants read,

*“The employee does not always see areas for change. The employee uses motivation methods that are not always effective but are generally focused on achieving the goal. In general, the employee does not create conflict situations.*

In the immoral condition, we change the information to indicate immorality:

*“The employee does not always see areas for change. The employee does not set a good example and does not motivate other employees. The employee was found to alter the job sheet to hide being late in the workplace.”*

In the moral condition, we change the information to indicate morality:

*“The employee does not always see areas for change. The employee sets a good example and motivates other employees. The employee always admits to being late in the workplace and never alters the job sheet.”*

Next, participants reported their attitude toward the target and then evaluated the target’s moral character.

### Measures

*Attitudes.* toward the target person were measured with two items: “I like this person” and “I would like to meet this person in the future” Participants indicated to what extent they agree with each of the statements using a 7-point scale from 1 = *definitely not* to 7 = *definitely yes* (α = 0.85, *M* = 4.28, *SD* = 1.41).

*Moral character judgments.* of the target person were measured with a 20-item version of the Agency-Communion-Inventory, which included moral character judgments^51^. Participants indicated the extent to which they agreed that the target person has five specific moral traits (trustworthy, fair, just, considerate, reliable) using a 7-point scale from 1 = *definitely not* to 7 = *definitely yes* (α = 0.93, *M* = 4.34, *SD* = 1.55).

### Results

#### Attitudes

Participants liked the target person more in the mimicry condition (*M* = 4.92, *SD* = 1.36) than in the no mimicry condition (*M* = 3.66, *SD* = 1.16), *F*(1, 647) = 185.32, *p* < 0.001, ω^2^_*p*_ = 0.22, 95% CI [0.17, 0.28]. Moreover, when the target’s past behavior was moral, participants liked the target to a higher degree (*M* = 4.75, *SD* = 1.20) than in the control condition (*M* = 4.43, *SD* = 1.35) and when the target’s past behavior was immoral (*M* = 3.65, *SD* = 1.45), *F*(2, 647) = 49.55, *p* < 0.001, ω^2^_p_ = 0.13, 95% CI [0.08, 0.18]. The interaction effect was nonsignificant, *F*(2, 647) = 1.85, *p* = 0.158, ω^2^_*p*_ = 0.00, 95% CI [0.00, 0.01].

#### Moral character judgments

**Figure 1:**
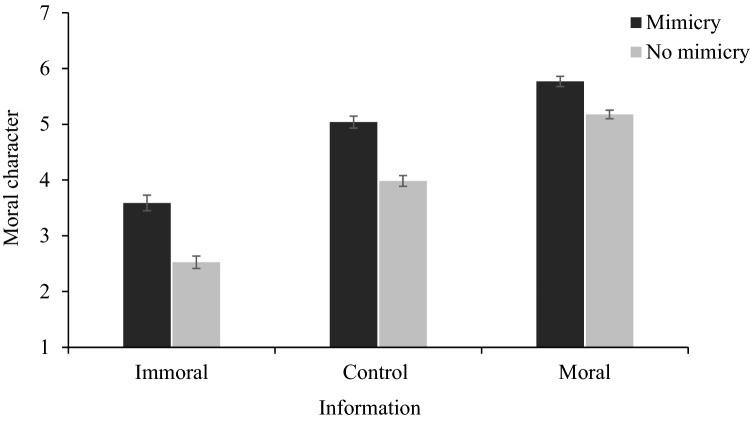
Mean moral character judgments as a function of the moral information and mimicry manipulation. Higher scores indicate more positive assessments of moral character. The error bars represent one standard error.

To test whether moral information would moderate the influence of liking on moral attributions, we have performed a 2 (attitude: mimicry vs. no mimicry) × 2 (information: moral vs. control vs. immoral) between-participants ANOVA. This analysis yielded a significant interaction of the two factors for the perception of moral character, *F*(2, 647) = 3.31, *p* = 0.037, ω^2^_p_ = 0.01, 95% CI [0.00, 0.02], (see Fig. 1).

Specifically, in the control condition, the target was perceived as being more moral when mimicked and less moral when not (M = 5.04, SD = 1.10 vs. M = 3.98, SD = 1.01), t(216) = 7.38, *p* < 0.001, *d* = 1.04, 95% CI [0.71, 1.28]. This effect was lower when the past behavior of the target was immoral, t(202.882) = 5.98, *p* < 0.001, *d* = 0.74, 95% CI [0.53, 1.09] and moral t(216) = 4.96, *p* < 0.001, *d* = 0.62, 95% CI [0.40, 0.94]. As we cannot compare if the difference between the size of effects is significant and therefore conclude if the liking bias was reduced or not, we decided to use the simple effects comparisons for the information manipulation at the mimicry and no-mimicry conditions separately. Since higher liking was produced by mimicry and liking biases moral character judgments, we believe that comparison of mimicry effect at each level of moral information allows us to conclude if the liking bias was reduced.

The simple effects were compared with the use of Games-Howell post-hoc comparison test, and all were significant at the level of *p* < 0.001. These comparison showed that judgments of moral character where lower in the immoral condition (*M* = 3.59, *SD* = 1.44) and higher in the moral condition (*M* = 5.77, *SD* = 0.95) in comparison to the control condition (*M* = 5.04 *SD* = 1.10), *F*(2, 318) = 94.12, *p* < 0.001, ω^2^ = 0.37, 95% CI [0.28, 0.45]. The same pattern of results, but with lower values, was observed in the no mimicry condition, *F*(2, 329) = 192.03, *p* < 0.001, ω^2^ = 0.54, 95% CI [0.46, 0.60], (see Table 1). Therefore, the simple effects for the mimicry condition corroborated that moral information had a significant impact on attributions of the target’s moral character.

**Table 1:** Means and standard deviations for the moral character judgments in Study 1.

Attitude	Information							
	Moral		Control		Immoral		Marginal	
	M	SD	M	SD	M	SD	M	SD
Mimicry	5.77	0.95	5.04	1.10	3.59	1.44	4.80	1.48
No mimicry	5.18	0.81	3.98	1.01	2.53	1.17	3.89	1.48
Marginal	5.47	0.93	4.51	1.18	3.05	1.41		

*M* and *SD* represents mean and standard deviation, respectively.

### Discussion

Study 1 provided initial support for the hypothesis that adding moral information about an already liked target would limit the influence of liking on moral character judgments. Although liking biased participants’ perception of the target’s moral traits, this perception was updated according to the provided moral information. Specifically, in the mimicry condition, the target was less moral when behaved immorally in work and more moral when behaved morally. However, in the no mimicry condition, the same but a stronger pattern of results emerged. Therefore, these results suggest that liking, even in the light of moral information, still biases moral character attributions.

We argue that moral character attributions triggered by liking are less diagnostic and less certain than moral character attributions triggered by moral information. As we could observe in Study 1, moral information updated moral character impressions triggered by liking. However, since moral information is more diagnostic and results in more certainty than liking, moral character attributions triggered by moral information should not be updated by liking. We directly addressed this hypothesis in Study 2.

## Study 2

In Study 2, we sought to extend the results of Study 1 by introducing morally relevant information about the target before the attitude induction. To this end, we used the same employee assessment form as in Study 1. However, we presented it before, inducing a positive attitude toward the target. Moreover, because of the COVID-19 pandemic at the time of the study (which stopped lab-based experiments), we had to adjust the attitude induction to the online environment. Therefore, we used a bogus stranger paradigm from the study by Sprecher^52^. Specifically, based on answers to a self-descriptive questionnaire, we convinced participants that the target person has similar or dissimilar preferences to their preferences. This attitude induction has at least one advantage over the attitude manipulation used in Study 1.

One could argue that mimicry manipulation is a manipulation of cooperation as the target person repeats the participant’s facial expressions. Because research suggests that various forms of cooperative behaviors are perceived as morally right^53^, mimicry manipulation could act as moral manipulation. If this is the case of Study 1, then mimicry manipulation could already reveal some information about the target’s morality, thus reducing the impact of moral information manipulation on the target’s moral character attributions. The current manipulation of similarity in preferences (e.g., coffee vs. tea or Mac vs. PC) is more subtle, less biased, and devoid of any relevance to morality.

We expected that similarity (vs. dissimilarity) of preferences would result in higher (vs. lower) liking of the target and more favorable (vs. less favorable) judgments of the target’s moral character. However, we also predicted that providing moral information before induction of liking would moderate the effect of the liking bias on moral character judgments. Specifically, we assumed that if moral information influences judgments of moral character less strongly than liking, then we would observe more favorable character judgments of a similar than dissimilar target person. However, if moral information has a stronger impact on character judgments than liking, we should observe a reduction or even elimination of the liking bias in both immoral and moral conditions. We preregistered the hypotheses for this study at https://aspredicted.org/fi8yb.pdf.

### Method

#### Participants and procedure

According to the results of similarity manipulation on liking/disliking found in Sprecher^52^, the effect was *d* = 0.92. Therefore, using the G*Power calculator^49^, we calculated that with the power of 0.95, we need a total sample size of 546 participants (91 per condition) to obtain the same effect size. Considering the possible exclusions, we sought to recruit at least 600 people, 100 for each condition. We achieved the planned sample size. We recruited 601 British participants using Prolific Academic (301 women; mean age = 40.68 years, *SD* = 13.95) to participate in an online study about the social perception of other people in their workplace. Both factors were manipulated between participants. Based on a sensitivity power analysis, this sample size provides 0.80 power to detect an effect size of *f*^2^ = 0.13.

For the attitude induction, we used a similarity/dissimilarity manipulation from the study of Sprecher^52^. Specifically, we asked participants to complete a preference form in which they had to answer 17 questions about their preferences (e.g., “Which do you prefer?—reality show vs. sitcom”, “Which best describes you?—dreamer vs. doer”; see the Supplement for the full list of questions). When participants completed the preference form, we told them that a special algorithm would draw two bits of information about a random employee from a UK company. The first information presented a short assessment form used in Study 1. The second information showed the preference questionnaire completed by the employee. Based on the random manipulation, participants saw either that the employee’s 14 out of 17 answers were the same as their answers (the similar preferences condition) or that the 14 out of 17 responses were the opposite (the dissimilar preferences condition). Next, participants answered the same questions as in Study 1 regarding the attitude toward the employee and the employee’s moral character.

### Measures

*Attitudes.* toward the target person were measured as in Study 1 (α = 0.86, *M* = 4.03, *SD* = 1.17).

*Moral character judgments.* of the target person were measured with the same five moral traits as used in Study 1 (α = 0.92, *M* = 3.94, *SD* = 1.29).

### Results

#### Attitude

As predicted, the target person who had similar preferences to participants was liked by them more (*M* = 4.30, *SD* = 1.16) than the target person who had dissimilar preferences (*M* = 3.77, *SD* = 1.13), *F*(1, 595) = 30.30, *p* < 0.001, ω^2^_*p*_ = 0.05, 95% CI [0.02, 0.08]. Corroborating the results of Study 1, participants liked the target person stronger in the moral condition (*M* = 4.51, *SD* = 0.96) than in the control condition (*M* = 4.22, *SD* = 1.04) and in the immoral condition (*M* = 3.38, *SD* = 1.21), *F*(2, 595) = 58.76, *p* < 0.001, ω^2^_p_ = 0.16, 95% CI [0.11, 0.22]. The interaction effect was nonsignificant, *F*(2, 595) = 0.59, *p* = 0.556, ω^2^_*p*_ = − 0.00, 95% CI [0.00, 1.00].

#### Moral character judgments

To test whether moral information would moderate the influence of liking-disliking on moral character judgments, we have performed a 2 (preference: similar vs. dissimilar) × 3 (information: moral vs. control vs. immoral) between-participants ANOVA. This analysis yielded a significant main effect of preferences, with moral character of the target who had preferences similar to participants judged as more moral (*M* = 4.08, *SD* = 1.28) than character of the target with dissimilar preferences (*M* = 3.80, *SD* = 1.28), *F*(1, 595) = 6.33, *p* = 0.012, ω^2^_p_ = 0.01, 95% CI [0.00, 0.03]. The main effect of the target’s moral behavior was also significant with the target being perceived as more moral in the moral behavior condition (*M* = 4.86, *SD* = 0.94) than in the control condition (*M* = 4.19, *SD* = 0.83) as well as in the immoral behavior condition (*M* = 2.77, *SD* = 1.06), *F*(2, 595) = 251.91, *p* < 0.001, ω^2^_p_ = 0.46, 95% CI [0.39, 0.51]. However, the interaction between the preference and moral behavior manipulation was nonsignificant, *F*(2, 595) = 0.38, *p* = 0.683, ω^2^_*p*_ = − 0.00, 95% CI [0.00, 1.00], (see Table 2).

**Table 2:** Means and standard deviations for the moral character judgments in Study 2.

Preference	Information							
	Moral		Control		Immoral		Marginal	
	M	SD	M	SD	M	SD	M	SD
Similar	4.91	0.95	4.33	0.83	2.88	1.10	4.08	1.28
Dissimilar	4.80	0.92	4.05	0.81	2.67	1.02	3.80	1.28
Marginal	4.86	0.94	4.19	0.83	2.77	1.06		

*M* and *SD* represents mean and standard deviation, respectively.

### Discussion

Study 2 replicated Study 1 by demonstrating that the manipulation of preference similarity influenced participants’ attitudes toward the target and perception of the target’s moral character. The target was liked more and judged as having more moral character when the target’s preferences were similar to those of the participants. However, we did not find the interaction effect between preferences and moral information manipulation. Therefore, we cannot conclude if the liking bias was eliminated because moral information was presented before the attitude induction. Nevertheless, the size of the main effects suggests that moral information had a stronger impact on moral character judgments than interpersonal attitudes. Particularly, the effect size for the main effect of preferences was ω^2^_*p*_ = 0.01, while the effect size for moral information was 46 times bigger, ω^2^_*p*_ = 0.46.

The main effects comparison suggests that moral information had a more substantial impact on moral character inferences than interpersonal attitudes. This is a probable explanation for why we did not find the interaction effect. When people first get information about others’ moral past, later induction of liking or disliking does not lead to impression updating because moral information is more diagnostic and provides more certainty than interpersonal attitudes. However, to verify this hypothesis, we should manipulate in one study whether moral information appears before or after the attitude manipulation. Moreover, Bayesian inference models^9^ and the model of social uncertainty^54^ suggest that certainty about moral character inferences may impact the probability of impression updating, which could explain the effects observed in Study 1 and Study 2. We addressed these points in the final study.

## Study 3

In Study 3, we pursued two goals. First, we sought to replicate the results of Study 2 by manipulating whether moral information would be presented before or after the attitude induction. Therefore, we once more used an employee assessment form from the immoral condition of Studies 1 and 2. We decided to use the immoral condition only as immoral information has a more substantial impact on impression updating than moral information^39^, and beliefs about the morality of bad (vs. good) agents are more uncertain and amenable to updating^40^. We measured moral character judgments before and after the moral information presentation to test whether the moment of moral information introduction leads to impression updating. We assumed that participants would update their moral character judgments when moral information would be presented after, but not before, the attitude manipulation.

Second, we investigated whether certainty could be a potential driving force behind the effects found in Study 1 and Study 2. To this end, we measured participants’ certainty regarding their moral character judgments. We hypothesized that participants would be less certain about the target’s moral character after the attitude induction than after the introduction of moral information. Low certainty would lead to impression updating based on the incoming later and more certain moral information. In contrast, as moral information would produce more substantial certainty about the target’s moral character, incoming later attitude similarity or dissimilarity would not lead to impression updating because of a lower certainty value. Therefore, we assumed that the certainty of participants’ change in moral character judgments would mediate the moral character impression updating.

### Method

#### Participants and design

The main effects of attitude manipulation were respectively ω^2^_*p*_ = 0.22 for Study 1 and ω^2^_*p*_ = 0.05 for Study 2. Using G*Power^49^, we estimated the target sample size to be *N* = 29 (assuming a power of 0.95, two-tailed) to replicate this effect. Because we expected a 50% attenuation when the moral information would be presented before the attitude manipulation, we increased the sample size 14 times, which resulted in a target of 406 participants. We managed to collect data from 398 British participants using Prolific Academic (198 women; mean age = 40.52 years, *SD* = 13.47) to participate in an online study about the social perception of people in their workplace. Based on a sensitivity power analysis, this sample size provides 0.80 power to detect an effect size of *f*^2^ = 0.04.

The experiment employed a 2 (attitude: positive vs. negative) × 2 (the sequence: moral information first vs. moral information second) design with both factors manipulated between participants. The attitude manipulation was the same as in Study 2. For the moral information presentation, we used the employee assessment form from Studies 1 and 2 but only the immoral version where the supervisor mentioned the target’s immoral behavior in the workplace. Thus, we manipulated whether the information about the target’s immoral behavior was presented before or after the attitude induction and whether the attitude was positive (similar preferences) or dissimilar (dissimilar preferences).

#### Procedure

As in previous studies, we asked participants to report their attitude toward the target and then to judge the target’s moral character. However, in contrast to previous studies, attitude and moral character judgments were measured twice after introducing moral information and after attitude induction. For example, participants first saw the employee assessment form in the positive attitude and the moral information first condition. They then indicated their attitude and character judgments of the target. Afterward, they learned that the target’s preferences are similar to their own and then once more indicated their attitude and moral character judgments. In addition, we asked participants to what extent they were confident in their answers regarding both their attitude and moral character judgments.

### Measures

*Attitudes* toward the target person were measured as in Study 2 but two times (Time 1: α = 0.88, *M* = 3.91, *SD* = 1.36; Time 2: α = 0.89, *M* = 3.21, *SD* = 1.25).

*Moral character judgments* of the target person were measured with the same five moral traits as in Study 2 but two times (Time 1: α = 0.94, *M* = 3.61, *SD* = 1.36; Time 2: α = 0.94, *M* = 2.89, *SD* = 1.18).

*Moral character judgments’ certainty* was measured with a single item. Participants were asked to report separately for each moral trait how certain were they with their answers on a 9-point sliding scale with anchors 0 = *not at all certain* to 8 = *completely* (Time 1: α = 0.93, *M* = 4.76, *SD* = 1.64; Time 2: α = 0.93, *M* = 5.17, *SD* = 1.34).

### Results

#### Attitudes

**Figure 2:**
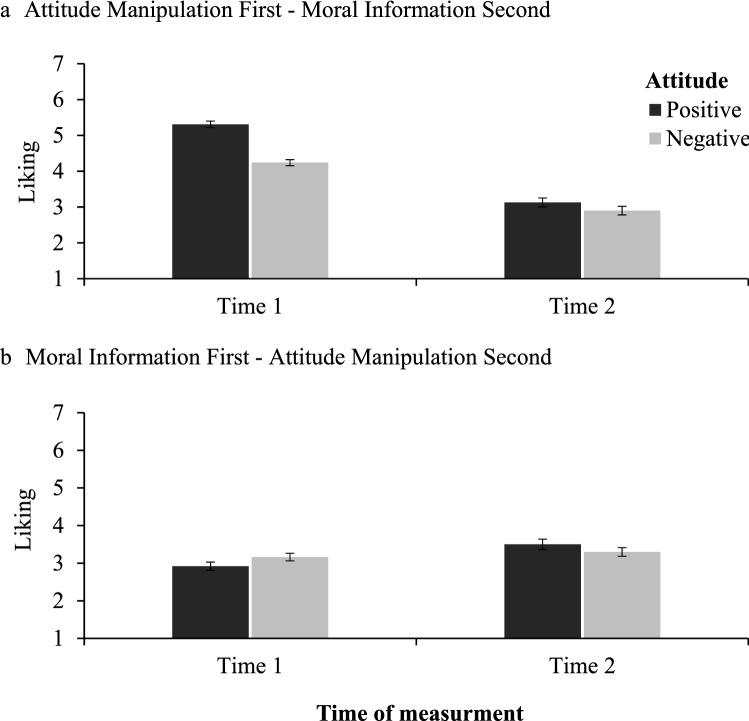
Mean liking judgments at Time 1 and Time 2 as a function of the sequence and attitude manipulation. Higher scores indicate more positive judgments of moral character. The error bars represent one standard error.

Similar as in Study 2 the target person who had similar preferences to participants was liked more (*M* = 3.72, *SD* = 1.13) than the target person who had dissimilar preferences (*M* = 3.40, *SD* = 0.89), *F*(1, 394) = 11.03, *p* < 0.001, ω^2^_*p*_ = 0.02, 95% CI [0.00, 0.06]. Moreover, participants liked the target more when the moral information was presented after the attitude induction (*M* = 3.90, *SD* = 0.91) and less when it was presented before the induction (*M* = 3.23, *SD* = 1.03), *F*(1, 394) = 50.74, *p* < 0.001, ω^2^_*p*_ = 0.11, 95% CI [0.06, 0.17]. The interaction effect of the attitude, sequence and time was also significant, *F*(1, 394) = 29.45, *p* < 0.001, ω^2^_*p*_ = 0.04, 95% CI [0.01, 0.09], (see Fig. 2).

At Time 1, when moral information was presented after the attitude induction, participants liked more the target who had similar preferences than the target who had dissimilar preferences (*M* = 5.31, *SD* = 0.92 vs. *M* = 4.24, *SD* = 0.84), *t*(198) = 8.62, *p* < 0.001, *d* = 0.61, 95% CI [0.46, 0.76]. At Time 2, the effect of preferences on liking was eliminated, *t*(198) = 1.34, *p* = 0.180, *d* = 0.10, 95% CI [− 0.04, 0.23]. Similar, when moral information was presented before the attitude induction, at Time 2 there was no effect of preferences on liking, *t*(189.17) = 1.12, *p* = 0.264, *d* = 0.08, 95% CI [− 0.06, 0.22]. However, one interesting pattern of results emerged. Particularly, we found that the similarity of preferences changed participants’ attitudes toward the target, Time 1: (*M* = 2.92, *SD* = 1.09) vs. Time 2: (*M* = 3.50, *SD* = 1.39), *t*(98) = 5.52, *p* < 0.001, *d* = 0.55, 95% CI [0.34, 0.77]. This effect did not occur for the dissimilarity of preferences, *t*(98) = 1.20, *p* = 0.234, *d* = 0.12, 95% CI [− 0.08, 0.32].

#### Moral character judgments

To test whether the sequence of presenting the moral information would influence the liking bias in moral character judgments, we performed a 2 (attitude: positive vs. negative) × 2 (sequence: moral information first vs. second) × 2 (time of moral character judgment: time 1 vs. time 2) mixed-model ANOVA with the two first factors between and the third within participants. This analysis yielded a significant main effect of sequence, with the target being judged as more moral when moral information was presented after the attitude induction (*M* = 3.60, *SD* = 0.72) and less moral when moral information was presented before the induction (*M* = 2.89, *SD* = 0.93; *F*(1, 394) = 73.99, *p* < 0.001, ω^2^_p_ = 0.15, 95% CI [0.09, 0.22]). The main effect of attitude induction was nonsignificant, *F*(1, 394) = 1.41, *p* = 0.235, ω^2^_*p*_ = 0.00, 95% CI [0.00, 0.02]. Finally, the interaction effect of the attitude, sequence and time was also significant, *F*(1, 394) = 6.53, *p* = 0.011, ω^2^_*p*_ = 0.01, 95% CI [0.00, 0.03].

**Figure 3:**
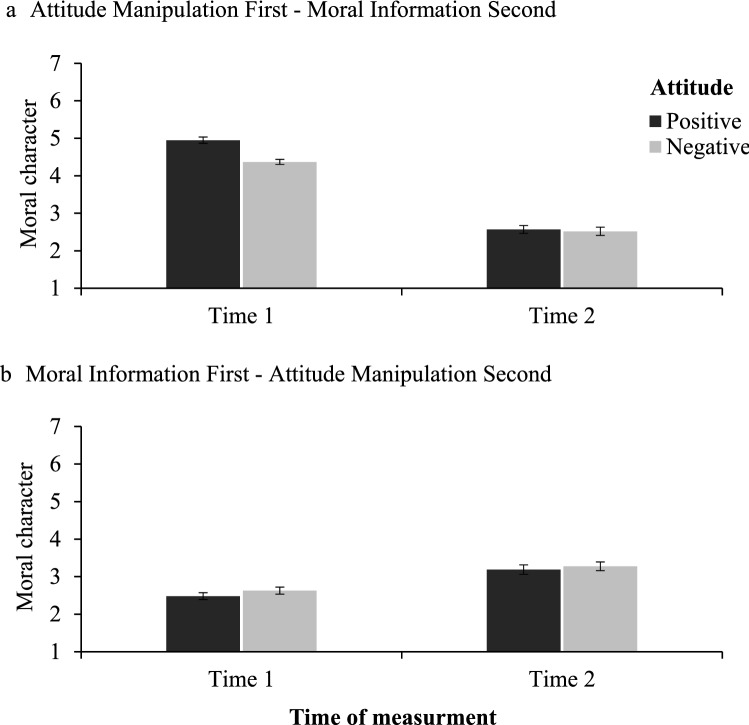
Mean moral character judgments at Time 1 and Time 2 as a function of the sequence and attitude manipulation. Higher scores indicate more positive judgments of moral character. The error bars represent one standard error.

Corroborating results of Study 1, when moral information was presented after the attitude induction at Time 1 participants judged the target who had similar preferences as more moral (*M* = 4.95, *SD* = 0.86) than target who had dissimilar preferences (*M* = 4.37, *SD* = 0.67; *t*(187.63) = 5.30, *p* < 0.001, *d* = 0.38, 95% CI [0.23, 0.52]). At Time 2, the impact of attitude on moral character judgments was eliminated, *t*(198) = 0.33, *p* = 0.743, *d* = 0.02, 95% CI [− 0.12, 0.16]. Moreover, when moral information was presented before the attitude induction, the liking bias was eliminated at both Time 1, *t*(196) = 1.14, *p* = 0.256, *d* = 0.08, 95% CI [− 0.06, 0.22] and Time 2, *t*(196) = 0.52, *p* = 0.602, *d* = 0.04, 95% CI [− 0.10, 0.18], (see Fig. 3).

Interestingly, and in contrast to Study 2, we also found that participants updated their moral character judgments when moral information was presented before the attitude induction. Specifically, positive attitude induction, Time 1: (*M* = 2.48, *SD* = 0.90) vs. Time 2: (*M* = 3.19, *SD* = 1.24), *t*(98) = 7.01, *p* < 0.001, *d* = 0.70, 95% CI [0.48, 0.92], and negative attitude induction, Time 1: (*M* = 2.63, *SD* = 0.92) vs. Time 2: (*M* = 3.28, *SD* = 1.15), *t*(99) = 5.96, *p* < 0.001, *d* = 0.60, 95% CI [0.38, 0.81] improved the perception of the target’s moral character judgments. We discuss this result more extensively in the Discussion.

#### Updating of moral character impression

To test to what degree updating of moral character impression depends on the moment of moral information presentation, we first computed an index of moral character impression updating. To this end, we subtracted the score for moral character judgment reported by participants after the second manipulation from the score reported after the first manipulation (Time 1–Time 2). Therefore, the greater the index—either positive or negative—the more significant the impression change in both sequence conditions.

**Figure 4:**
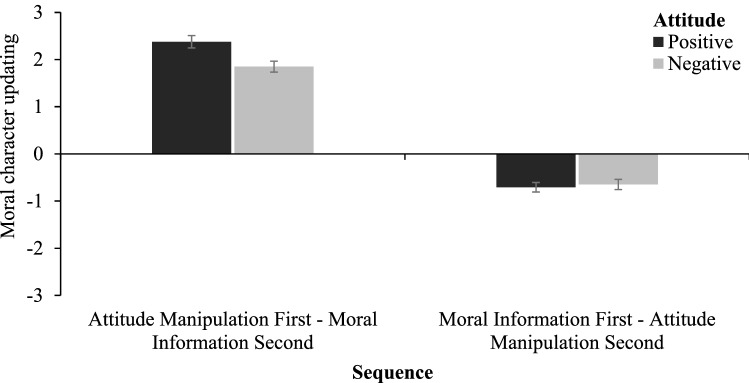
Mean index of updating impressions of moral character as a function of the sequence and attitude manipulation. Higher scores in the positive or negative direction indicate a greater impression change. The error bars represent one standard error.

Using the index of moral character impression updating, we have performed a 2 (attitude: positive vs. negative) × 2 (sequence: moral information first vs. second) between-participants ANOVA (see Fig. 4). This analysis yielded a main effect of attitude, *F*(1, 394) = 4.18, *p* = 0.041, ω^2^_p_ = 0.01, 95% CI [0.00, 0.03], with the index of moral character impression updating greater in the positive than negative attitude (*M* = 0.84, *SD* = 1.93 vs. *M* = 0.61, *SD* = 1.68). The main effect of sequence was also significant, *F*(1, 394) = 591.89, *p* < 0.001, ω^2^_p_ = 0.60, 95% CI [0.53, 0.66], with the index of updating was greater when moral information was presented after than before the attitude induction (*M* = 2.11, *SD* = 1.26 vs. *M* = − 0.68, *SD* = 1.04). Finally, the interaction between the attitude and sequence was also significant, *F*(1, 394) = 6.53, *p* = 0.011, ω^2^_p_ = 0.01, 95% CI [0.00, 0.05]. Further analysis revealed that when moral information was presented after the attitude induction the index of moral character impression updating was greater in the positive (*M* = 2.38, *SD* = 1.30) than negative (*M* = 1.85, *SD* = 1.16) attitude condition, *t*(198) = 3.02, *p* = 0.003, *d* = 0.43, 95% CI [0.15, 0.71]. In contrast, when moral information was presented before the attitude induction there was no difference between positive and negative attitude conditions, *t*(196) = 0.40, *p* = 0.694, *d* = 0.08, 95% CI [− 0.54, 0.82].

#### Certainty of moral character judgments

**Figure 5:**
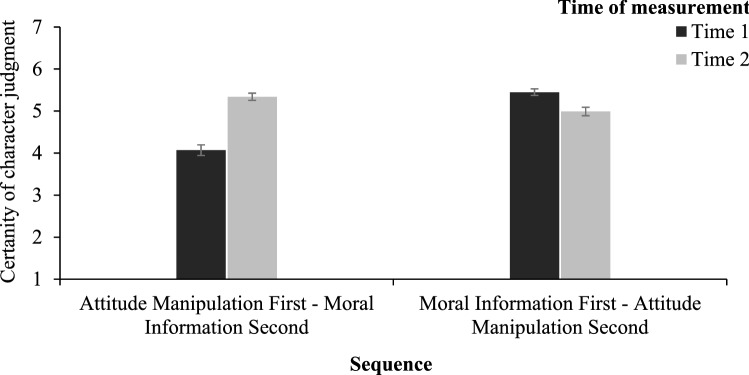
Mean certainty of moral character judgments as a function of the sequence and the time of certainty measurement. Higher scores indicate greater certainty. The error bars represent one standard error.

To test the hypothesis that moral character judgments based on attitudes are less certain than judgments based on moral information, we run a 2 (sequence: moral information first vs. second) × 2 (time of certainty judgment: time 1 vs. time 2) mixed-model ANOVA with the first factor between and the second within participants on the estimates of certainty of moral character judgment (see Fig. 5). This analysis revealed an interaction between the sequence and the time of certainty measurement, *F*(1, 394) = 140.53, *p* < 0.001, ω^2^_p_ = 0.15, 95% CI [0.09, 0.21]. As expected, at Time 1 the moral character certainty was lower after the attitude induction (*M* = 4.07, *SD* = 1.79) than after the moral information introduction (*M* = 5.45, *SD* = 1.11; *t*(332.91) = 9.32, *p* < 0.001, *d* = 0.94, 95% CI [0.73, 1.14]. At Time 2, the certainty was higher after the moral information presentation (*M* = 5.34, *SD* = 1.23) than after the attitude induction (*M* = 4.99, *SD* = 1.42; *t*(387.38) = 2.62, *p* = 0.009, *d* = 0.26, 95% CI [0.07, 0.46]. Moreover, when the attitude was induced first and moral information was presented as second, certainty went up, *t*(199) = 10.04, *p* < 0.001, *d* = 0.71, 95% CI [0.55, 0.86], but moved down when moral information was presented first and attitude was induced as second, *t*(199) = 6.08, *p* < 0.001, *d* = 0.43, 95% CI [0.29, 0.58].

#### Certainty changes as a mediator

To test whether changes in the certainty of moral character judgments mediate the updating of moral character impressions, we first computed an index of moral character certainty change. To this end, we subtracted the score for moral character judgment certainty reported by participants after the first manipulation from the score reported after the second manipulation. Afterward, we run mediation Model 4 in PROCESS macro proposed by Hayes^54^ with the moral information sequence manipulation coded as -1 (attitude first, moral information second) vs. 1 (moral information first, attitude second), with the index of moral character impression updating as a depended variable and the index of moral character certainty change serving as a mediator. The indirect effect of the moral information sequence manipulation on the index of moral character impression updating appeared significant (we used attitude manipulation as a covariant), *B* = − 0.18, S*E* = 0.03, 95% CI = [− 0.25, − 0.11], and therefore corroborated that change in participants’ moral character certainty mediated the influence of moral information sequence on moral character impression updating.

### Discussion

Study 3 showed that the moment of moral information presentation (before or after the attitude induction) had a significant impact on the liking bias in the perception of moral character. Specifically, we found evidence confirming that liking (vs. disliking) influenced moral character judgments when moral information about the target person was presented after the attitude induction. However, the liking bias was eliminated when moral information preceded attitude induction. Moreover, using impression updating methodology, Study 3 demonstrated that participants updated their moral character impressions more when moral information was presented after (vs. before) the attitude induction.

Therefore, these results confirm that moral information, in contrast to attitudes, has a more substantial impact on moral character inferences. As a result, moral information leads to significant impression updating when moral character judgments derive from interpersonal attitudes. In contrast, impression updating is three times smaller when interpersonal attitudes update moral character judgments grounded in moral information. Nevertheless, in contrast to Study 2, we found that after the moral information presentation, the attitude induction updated participants’ moral character impressions. Nonetheless, we argue that this effect is rather driven by a more general effect than the liking bias.

First, only a positive induction of attitude resulted in a change of liking, while a negative induction of attitude had no effect. However, the perception of moral character was improved after both positive and negative inductions of attitude. Second, according to the liking bias research^3,27^, moral character judgments follow interpersonal attitudes. Thus, if the liking bias would drive the observed effect, we should observe higher moral character judgments in the positive attitude condition and lower ones in the negative attitude condition.

Therefore, we argue that the observed result is probably a byproduct of a more general effect (e.g., a revelation of additional moral information). For example, research has shown that seemingly apolitical preferences become politicized^55^. Moreover, since ideology correlates with moral convictions^56^, preference manipulation may have moral undertones (similar preferences = moral, dissimilar preferences = immoral).

Study 3 demonstrated the potential driving force behind the effects found in Study 1 and Study 2. As predicted, participants showed less certainty when their moral judgments had been based on interpersonal attitudes and more certainty when they had been nestled in moral information. Further analysis confirmed that the certainty of participants’ changes in moral character judgments mediated updating moral character impressions. Therefore, this result provided evidence that confidence, primarily the span of certain changes in the light of incoming attitudinal or moral information, may explain why moral information leads to more substantial impression updating than attitudes. Corroborating assumptions of Bayesian inference models^9^ and the model of social uncertainty^46^, moral information, in contrast to attitudes, brings more certainty about moral character inferences and, as a result, attitudinal influences on moral character attributions are updated by moral information. Still, attitudinal influences merely update moral character attributions nestled in moral information.

## General discussion

This research investigated how interpersonal attitudes and morally relevant information influence moral character judgments. In addition, we tested the psychological mechanism underlying updating process of moral character impressions. We demonstrated that liking elicited by mimicry (Study 1) and preference similarity (Studies 2 & 3) influence moral character judgments. Therefore, we corroborated previous findings^3,27^, demonstrating the subtle influence of interpersonal attitudes on moral character judgments. More importantly, we found evidence that morally relevant information reduced (Studies 1 & 3) and eliminated (Studies 2 & 3) the liking bias in moral character judgments. Finally, we confirmed (Study 3) that moral information updates moral character judgments triggered by interpersonal attitudes to a greater extent than interpersonal attitudes update moral character judgments based on moral information. As we confirmed in Study 3, the driving force behind this difference was the variation in the certainty of moral character judgments.

On the one hand, these results demonstrate how negativity bias and liking bias interact in impression updating. On the other hand, the results confirm models of social uncertainty^46^ and Bayesian inference^9^ because both models argue that certainty about moral character inferences may impact the probability of impression updating. Study 1 demonstrated that moral information, especially the negative one, about the target’s past behavior introduced after the mimicry manipulation reduced, although not eliminated, the liking bias in moral character judgments. Study 2 found evidence that the liking bias in character judgments was eliminated when moral information was introduced before the attitude induction. Study 3 evidenced that liking bias was reduced when moral information came second (after attitude induction) but was eliminated when moral information came first (before attitude induction).

We established that participants’ certainty regarding moral character judgments explained when and how morally relevant information moderates the liking bias. We found that participants were less certain about their moral character judgments based on attitudes than morally relevant information. As a result, moral information updated impressions triggered by attitudes to more extent than attitudes. Further analysis confirmed that the change in participants’ certainty was larger when moral information revised moral character judgments based on attitudes than when attitudes revised moral character judgments based on moral information. This change explained why moral information presentation after (vs. before) attitudes reduced (vs. eliminated) the impression updating process and, therefore, the liking bias in moral character judgments.

By systematically examining how interpersonal attitudes and moral information impact moral character judgments, we built on and extended the past work in moral and social cognition. Past studies have focused on either how attitudes influence moral character judgments^2–5^ or how morality impacts impression updating^35^, perceptions of trustworthiness of social partners^57^, and leaders^58^. This work examined how liking (vs. disliking) and morality (vs. immorality) shape moral character inferences. Thus, we demonstrated that moral information impacts the liking bias in moral character judgments, but whether the liking bias would be limited or eliminated depends on the moment of moral information presentation.

These results extend past research on impression updating^7,8,36^, corroborate The Moral Primacy Model of impression development^6^, and confirm that morality has a significant impact on impression change^35^. Specifically, gathered evidence suggests that morality strongly updates moral character impressions biased by liking, but interpersonal liking updates moral character impressions based on morality only slightly. At least two mechanisms can explain this effect.

According to the model of social uncertainty^46^, social stimuli may trigger automatic and controlled inferences about others’ traits (e.g., morality), narrowing potential predictions about others’ behavior and helping people solve social uncertainty. Because people infer moral character fast and without effort^28,29^ and moral judgments are produced mainly by intuitive processes^59^, there is a high probability that automatic inferences are the primary mechanism explaining how interpersonal attitudes impact moral character judgments. This could explain our findings as morally relevant information should trigger more controlled inferences about others’ moral character than attitudes. However, this mechanism was not tested directly so we will raise this issue in the limitation section.

The second explanation is based on Bayesian inference models. Specifically, Crockett et al.^9^ argue that weak prior beliefs about others’ moral character are more prone to change than strong prior beliefs. Corroborating this assumption, we first demonstrated that people’s certainty about others’ moral character based on attitudes is weaker than certainty based on moral information. Later we confirmed that the span of change in participants' certainty explained why moral information but not attitudes lead to impression updating. Therefore, we found evidence confirming Bayesian inference models^9^, models of social uncertainty^46^, and research regarding certainty about the morality of good and bad agents^40^. We believe that present results contribute to a better understanding of the role of social uncertainty in moral character inferences.

Our work also extends recent research on conditions that reduce the attitudinal influences on moral character attributions. Specifically, while past work showed that the liking bias in moral character judgments could be attenuated with education on biases in social cognition or eliminated with accountability^27^, we demonstrated that introducing morally relevant information about the target’s past could be yet another successful technique helping people debias their character judgments contaminated by interpersonal attitudes. Therefore, we found further evidence suggesting that the influence of misleading intuitions on moral character judgments could be reduced or even eliminated when more controlled processing is required to generate these judgments.

## Limitations, implications, and future directions

We acknowledge that our work has several limitations that might warrant future research. First, even though our samples represent equally men and women from Poland (Study 1) and the UK (Study 2 and 3), students (Study 1) and the general population (Studies 2 and 3) presented results and, therefore, the generalizability of our findings is limited to people who live in Western, educated, industrialized, rich, and democratic (WEIRD) nations^60^. Future research could address this limitation by examining if cultural differences moderate the attitudinal influences on moral character inferences.

Second, past evidence has suggested that such factors as self and group interests or attitudes bias moral judgments because of automatically arising intuitions^10^. These assumptions were not, to date, tested directly. Correspondingly with the model of social uncertainty^46^ and the premises of dual-process models in social^12^ and moral cognition^11^, we suspect that interpersonal attitudes impact moral character judgments via a mechanism of automatic inferences. In contrast, moral information should affect moral character judgments via more controlled inferences.

As research investigating the role of controlled processing on moral judgments has so far focused either on individual differences or abilities in cognitive style^61^, in the future, we should establish what role automatic and controlled processes play in the influence of attitudes on moral judgments. For example, future research could use manipulations such as cognitive load, time pressure, or priming to establish to what extent attitudinal influences on moral judgments are driven by automatic (vs. controlled) processing.

Third, future research may answer the question about the mechanism underpinning the liking bias in moral character judgments. On the one hand, this mechanism may be linked to automatic and controlled inferences in impression updating. On the other hand, the influence of attitudes on moral character judgments may depend on specific cognitive mechanisms. For example, because research has found evidence for a strong correlation between liking and morality^62^, future research could investigate whether people hold associations between liking and judging someone as moral as well as under which conditions (e.g., lack of cognitive resources) these associations become stronger or weaker. Another promising avenue of research could test whether the striving for cognitive consistency^63^ may explain the link between attitudes and moral judgments. For example, a study may test whether people judge others they like as moral to avoid inconsistency between liking and moral judgments of the same object.

Finally, there is an alternative explanation for the results, which future work may address. One could argue that people judge similar (vs. dissimilar) people as moral (vs. immoral) because they use themselves as a reference point. If I am moral and this person is like me, then it must also be moral. Thus, moral information, which is more diagnostic than attitudes, updates moral character impressions. However, when moral information is provided first, especially about immorality, similarity (vs. dissimilarity) does not matter because people do not perceive themselves as immoral thus, from the very beginning, they do not identify themselves with the immoral characters. This alternative explanation could be tested by investigating to what extent moral (vs. immoral) information blocks comparisons with other people.

We believe our work might contribute to recent research that embeds moral judgments in a specific context (e.g., relationships). As a result, we challenge the mismatch between morality studied in a social vacuum and everyday morality based on different interpersonal relationships. For example, research had found evidence that people justified such acts as theft or sexual harassment when close others committed them^64^ or judged harmful behavior as less unethical when their siblings committed it than a stranger^65^. In the same vein, a different study has demonstrated that less morally good and trustworthy are agents who helped strangers instead of kin^66^.

This evidence aligns with the assumption of relationship regulation theory^67^, suggesting that moral judgments are embedded in our social-relational cognition and findings suggesting that people update moral impressions in response to ongoing social relationships^9^. Thus, whether an action would be judged as right or wrong or whether people would update their moral character impression heavily depends on the social-relation context in which it occurs. Given that attitudes strongly impact perceptions of moral character^2–5,27^, future research would do well by investigating how specific social and personal relations shape moral cognition.

## Conclusion

This paper systematically examined when and how moral information limits the influence of liking on moral character judgments. Therefore, we replicated prior findings of the liking bias^3^ and negativity bias^7,8^. Moreover, we demonstrated that moral information, apart from education and accountability^27^, could serve as another factor in helping people debias their moral character judgments. Finally, we found evidence suggesting that certainty is a potential psychological mechanism explaining why moral information leads to moral character impression updating. The presented results indicate that moral character inferences triggered by liking could be limited or even eliminated when morally relevant information about people in judgment is present. However, whether moral information would help people debias their moral character judgments mostly depends on interpersonal relationship with a judged person.

## Supplementary Information

Supplementary Information.

## Data Availability

All raw data files, analysis scripts, and materials used in this article are available for download from the Open Science Framework: https://osf.io/4znm7/?view_only=04355c07d5be4472add6d07f0c9bb41d.
