# Supplementary material for: The boundary conditions of the liking bias in moral character judgments
Source: Sci Rep. 2022 Oct 14;12:17217. doi: 10.1038/s41598-022-22147-7 (PMC9561316; doi:10.1038/s41598-022-22147-7)
Supplement: Supplementary file 1 — Supplementary Information. [file 41598_2022_22147_MOESM1_ESM.docx]

**Additional measures and analyses for:**

The Boundary Conditions of The Liking Bias in Moral Character Judgments

**Study 1**

**Warmth, competence, and assertiveness.** Past studies showed that when participants ascribed traits to the target person, the influence of liking on character impressions was limited to moral and warmth traits. However, when participants had to predict the target’s behavior, the influence of liking on character impressions spilled over into the competence traits (Bocian et al., 2018). Therefore, we sought to test if the influence of liking on character impression would be limited to moral traits or would impact the perception of traits related to competence as well. Moreover, we examined whether liking would shape participants’ perception of the target’s behavior as moral and competent.

**Measures.**

**Moral behavior judgments** of the target person were measured with two items: “The employee behaves fairly at work”, “The employee behaves honestly at work” Participants indicated to what extent they agreed with each of the statements using a 7-point scale from 1 = *definitely not* to 7 = *definitely yes* (α = .93, *M* = 4.50, *SD* = 1.81).

**Competence behavior judgments** of the target person were measured with two items: “The employee is effective at work”, and “The employee shows intelligence at work”. Participants indicated to what extent they agreed with each of the statements using a 7-point scale from 1 = *definitely not* to 7 = *definitely yes* (α = .78, *M* = 4.39, *SD* = 1.13).

**Trait perception** of the target person was measured with a 20-items version of the Agency-Communion-Inventory (Abele et al., 2016). Participants indicated to what extent they agreed that the target person is warm (α = .94, *M* = 4.32, *SD* = 1.59), assertive (α = .67, *M* = 3.66, *SD* = 1.00) and competent (α = .89, *M* = 4.38, *SD* = 1.04) using a 7-point scale from 1 = *definitely not* to 7 = *definitely yes.*

**Results**

The **zero-order correlations** are presented in Table S1. The attitude was positively correlated with all other variables.

| Table S1 |  |  |  |  |  |  |
| --- | --- | --- | --- | --- | --- | --- |
|  |  |  |  |  |  |  |
| *Table of Correlations for Main Variables (Study 1)* | | |  |  |  |  |
|  |  |  |  |  |  |  |
| Variables | 1 | 2 | 3 | 4 | 5 | 6 |
| 1. Attitude | - |  |  |  |  |  |
| 2. Moral behavior | .63*** | - |  |  |  |  |
| 3. Competence behavior | .61*** | .43*** | - |  |  |  |
| 4. Morality | .70*** | .87*** | .54*** | - |  |  |
| 5. Warmth | .73*** | .56*** | .56*** | .76*** | - |  |
| 6. Assertiveness | .13** | -0.04 | .35*** | .07 | .05 | - |
| 7. Competence | .56*** | .33*** | .80*** | .52*** | .58*** | .43*** |
|  |  |  |  |  |  |  |
| *Note. *** p* < .001*, ** p* < .01 | |  |  |  |  |  |

**Moral behavior**. Participants judged the target’s behavior as more moral in the mimicry condition (*M* = 4.81, *SD* = 1.74) than in the no mimicry condition (*M* = 4.20, *SD* = 1.82), *F*(1,647) = 38.23, *p* < .001, ω^2^_p_ = .05, 95% CI [.02, .09]. Additionally, participants judged the target’s behavior as more moral in the moral condition (*M* = 5.88, *SD* = 1.01) than in the immoral condition (*M* = 2.79, *SD* = 1.46) or the control condition (*M* = 4.82, *SD* = 1.31), *F*(2,647) = 1.85, *p* = .158, ω^2^_p_ = .52, 95% CI [.46, .57] which confirms that our moral information manipulation was effective. However, the interaction effect was nonsignificant, *F*(2,647) = 2.48, *p* = .084, ω^2^_p_ = .00, 95% CI [.00, .02].

**Competence behavior**. Participants judged the target’s behavior as more competent in the mimicry condition (*M* = 4.77, *SD* = 1.15) than in the no mimicry condition (*M* = 4.03, *SD* = 0.99), *F*(1,647) = 77.85, *p* < .001, ω^2^_p_ = .11, 95% CI [.06, .15]. Moreover, participants judged the target’s behavior as more competent in the moral condition (*M* = 4.56, *SD* = 1.07) than in the immoral condition (*M* = 4.31, *SD* = 1.19) but as the same level of competence as in the control condition (*M* = 4.30, *SD* = 1.13), *F*(2,647) = 4.28, *p* = .014, ω^2^_p_ = .01, 95% CI [.00, .03]. Nevertheless, the interaction effect was nonsignificant, *F*(2,647) = 1.94, *p* = .144, ω^2^_p_ = .00, 95% CI [.00, .01].

**Trait perception.** To test whether moral information would moderate the influence of liking on trait attributions different that moral ones, we performed a multivariate ANOVA with the attitude manipulation (mimicry vs. no mimicry) and the information about the target’s past behavior (moral vs. control vs. immoral) as a between-subjects factors. This analysis confirmed that effects of liking for remaining traits were nonsignificant (see the full trait analysis for Study 1 including moral traits).

**Full trait analysis (Study 1)**

| Table S2 |  |  |  |  |  |  |  |  |
| --- | --- | --- | --- | --- | --- | --- | --- | --- |
|  |  |  |  |  |  |  |  |  |
| *Means and standard deviations for the perception of moral traits as a function of a 2(attitude manipulation) X 3(moral behavior) design (Study 1)* | | | | | | | | |
|  |  |  |  |  |  |  |  |  |
|  | Behavior | | | | | |  |  |
|  | Moral | | Control | | Immoral | | Marginal | |
| Attitude manipulation | *M* | *SD* | *M* | *SD* | *M* | *SD* | *M* | *SD* |
| Mimicry | 5.77 | 0.95 | 5.04 | 1.10 | 3.59 | 1.44 | 4.80 | 1.48 |
| No mimicry | 5.18 | 0.81 | 3.98 | 1.01 | 2.53 | 1.17 | 3.89 | 1.48 |
| Marginal | 5.47 | 0.93 | 4.51 | 1.18 | 3.05 | 1.41 |  |  |
|  |  |  |  |  |  |  |  |  |
| *Note*. *M* and *SD* represents mean and standard deviation, respectively. | | | | | | |  |  |

| Table S3 |  |  |  | |  | |  | |  | | |  | |  |  |
| --- | --- | --- | --- | --- | --- | --- | --- | --- | --- | --- | --- | --- | --- | --- | --- |
|  |  |  |  | |  | |  | |  | | |  | |  |  |
| *Fixed-Effects* *ANOVA results for the perception of moral traits (Study 1)* | | | | | | | | | |  | | |  | | |
|  |  |  |  |  | |  | |  | | |  | | | |  |
| Predictor | Sum of Squares | *df* | Mean Square | *F* | | *p* | | ω^2^_p_ | | | ω^2^_p_  95% CI [LL, UL] | | | |  |
| (Intercept) | 12333.78 | 1 | 12333.78 | 10261.60 | | < .001 | |  | | |  | | | |  |
| Mimicry | 133.30 | 1 | 133.30 | 110.90 | | < .001 | | .14 | | | [.10, .20] | | | |  |
| Behavior | 642.52 | 2 | 321.26 | 267.28 | | < .001 | | .45 | | | [.39, .50] | | | |  |
| Mimicry × Behavior | 7.96 | 2 | 3.98 | 3.31 | | .037 | | .01 | | | [.00, .02] | | | |  |
| Error | 777.65 | 647 | 1.20 |  | |  | |  | | |  | | | |  |
|  |  |  |  |  | |  | |  | | |  | | | |  |
| *Note*. LL and UL represent the lower-limit and upper-limit of the partial ω^2^ confidence interval, respectively. | | | | | | | | | | | | | | | |

| Table S4 |  |  |  |  |  |  |  | |  | |  |
| --- | --- | --- | --- | --- | --- | --- | --- | --- | --- | --- | --- |
|  |  |  |  |  |  |  |  | |  | |  |
| *Means and standard deviations for the perception of warmth traits as a function of a 2(attitude manipulation) X 3(moral behavior) design (Study 1)* | | | | | | | | | | | |
|  |  |  |  |  |  |  |  | |  | |  |
|  | Behavior | | | | | |  | |  | |  |
|  | Moral | | Control | | Immoral | | Marginal | | | |  |
| Attitude manipulation | *M* | *SD* | *M* | *SD* | *M* | *SD* | *M* | | *SD* | |  |
| Mimicry | 5.74 | 1.04 | 5.38 | 1.24 | 4.58 | 1.54 | 5.24 | | 1.37 | |  |
| No mimicry | 3.98 | 1.32 | 3.40 | 1.04 | 2.90 | 1.08 | 3.43 | | 1.23 | |  |
| Marginal | 4.84 | 1.48 | 4.38 | 1.51 | 3.72 | 1.57 |  | |  | |  |
|  |  |  |  |  |  |  |  | |  | |  |
| *Note*. *M* and *SD* represents mean and standard deviation, respectively. | | | | | | | |  | |  | |

| Table S5 |  | |  | |  | | |  | |  | |  | |  | | |  |
| --- | --- | --- | --- | --- | --- | --- | --- | --- | --- | --- | --- | --- | --- | --- | --- | --- | --- |
|  |  | |  | |  | | |  | |  | |  | |  | | |  |
| *Fixed-Effects ANOVA results for the perception of warmth traits (Study 1)* | | | | | | | | | | | | | | | | |  |
|  |  | |  | |  | | |  | |  | |  | |  | | |  |
| Predictor | Sum of Squares | | *df* | | Mean Square | | | *F* | | *p* | | ω^2^_p_ | | ω^2^_p_  95% CI [LL, UL] | | |  |
| (Intercept) | 12249.73 | | 1 | | 12249.73 | | | 8193.37 | | < .001 | |  | |  | | |  |
| Mimicry | 533.77 | | 1 | | 533.77 | | | 357.02 | | < .001 | | .35 | | [.29, .41] | | |  |
| Behavior | 136.63 | | 2 | | 68.31 | | | 45.69 | | < .001 | | .12 | | [.08, .17] | | |  |
| Mimicry × Behavior | 2.69 | | 2 | | 1.35 | | | 0.90 | | .407 | | -.00 | | [.00, 1.00] | | |  |
| Error | 967.32 | | 647 | | 1.50 | | |  | |  | |  | |  | | |  |
|  |  | |  | |  | | |  | |  | |  | |  | | |  |
| *Note*. LL and UL represent the lower-limit and upper-limit of the partial ω^2^ confidence interval, respectively. | | | | | | | | | | | | | | | |  |  |
| Table S6 | |  | |  | |  |  | |  | |  | |  | |  | | |
|  | |  | |  | |  |  | |  | |  | |  | |  | | |
| *Means and standard deviations for the perception of competence traits as a function of a 2(attitude manipulation) X 3(moral behavior) design (Study 1)* | | | | | | | | | | | | | | | | | |
|  | |  | |  | |  |  | |  | |  | |  | |  | | |
|  | | Behavior | | | | | | | | | | |  | |  | | |
|  | | Moral | | | | Control | | | Immoral | | | | Marginal | | | | |
| Attitude manipulation | | *M* | | *SD* | | *M* | *SD* | | *M* | | *SD* | | *M* | | *SD* | | |
| Mimicry | | 4.84 | | 0.98 | | 4.74 | 0.96 | | 4.73 | | 1.01 | | 4.77 | | 0.98 | | |
| No mimicry | | 4.16 | | 0.84 | | 3.89 | 1.02 | | 3.97 | | 0.96 | | 4.01 | | 0.95 | | |
| Marginal | | 4.49 | | 0.97 | | 4.31 | 1.08 | | 4.34 | | 1.05 | |  | |  | | |
|  | |  | |  | |  |  | |  | |  | |  | |  | | |
| *Note*. *M* and *SD* represents mean and standard deviation, respectively. | | | | | | | | | | | | |  | |  | | |

| Table S7  *Fixed-Effects ANOVA results for the perception of competence traits (Study 1)* | | | | | | | |
| --- | --- | --- | --- | --- | --- | --- | --- |
| Predictor | Sum of Squares | *df* | Mean Square | *F* | *p* | ω^2^_p_ | ω^2^_p_  95% CI [LL, UL] |
| (Intercept) | 12571.84 | 1 | 12571.84 | 13514.22 | < .001 |  |  |
| Mimicry | 95.05 | 1 | 95.05 | 102.17 | < .001 | .13 | [.09, .19] |
| Behavior | 3.84 | 2 | 1.92 | 2.07 | .128 | .00 | [.00, .02] |
| Mimicry × Behavior | 0.80 | 2 | 0.40 | 0.43 | .650 | -.00 | [.00, 1.00] |
| Error | 601.88 | 647 | 0.93 |  |  |  |  |
|  | | | | | | | |
| *Note*. LL and UL represent the lower-limit and upper-limit of the partial ω^2^ confidence interval, respectively. | | | | | | | |

| Table S8 |  | |  | |  | |  | |  | |  | |  | |  | |  |  |
| --- | --- | --- | --- | --- | --- | --- | --- | --- | --- | --- | --- | --- | --- | --- | --- | --- | --- | --- |
|  | |  | |  | |  | |  | |  | |  | |  | |  | |  |
| *Means and standard deviations for the perception of the assertiveness traits as a function of a 2(attitude manipulation) X 3(moral behavior) design (Study 1)* | | | | | | | | | | | | | | | | | | |
|  | |  | |  | |  | |  | |  | |  | |  | |  | |  |
|  | | Behavior | | | | | | | | | | | |  | |  | |  |
|  | | Moral | | | | Control | | | | Immoral | | | | Marginal | | | |  |
| Attitude manipulation | | *M* | | *SD* | | *M* | | *SD* | | *M* | | *SD* | | *M* | | *SD* | |  |
| Mimicry | | 3.64 | | 1.05 | | 3.65 | | 1.04 | | 3.83 | | 1.05 | | 3.71 | | 1.05 | |  |
| No mimicry | | 3.56 | | 0.92 | | 3.51 | | 1.00 | | 3.79 | | 0.94 | | 3.62 | | 0.96 | |  |
| Marginal | | 3.60 | | 0.98 | | 3.58 | | 1.02 | | 3.81 | | 1.00 | |  | |  | |  |
|  |  | |  | |  | |  | |  | |  | |  | |  | |  |  |
| *Note*. *M* and *SD* represents mean and standard deviation, respectively. | | | | | | | | | | | | |  | |  | |  |  |

| Table S9 |  | |  | |  | |  | |  | |  | |  | | |  |
| --- | --- | --- | --- | --- | --- | --- | --- | --- | --- | --- | --- | --- | --- | --- | --- | --- |
|  |  | |  | |  | |  | |  | |  | |  | | |  |
| *Fixed-Effects ANOVA results for the perception of the assertiveness traits (Study 1)* | | | | | | | | | | | | | | |  |  |
|  |  | |  | |  | |  | |  | |  | |  | | |  |
| Predictor | Sum of Squares | | *df* | | Mean Square | | *F* | | *p* | | ω^2^_p_ | | ω^2^_p_  95% CI [LL, UL] | | |  |
| (Intercept) | 8760.51 | | 1 | | 8760.51 | | 8755.18 | | < .001 | |  | |  | | |  |
| Mimicry | 1.29 | | 1 | | 1.29 | | 1.29 | | .257 | | .00 | | [.00, .01] | | |  |
| Behavior | 7.01 | | 2 | | 3.51 | | 3.50 | | .031 | | .01 | | [.00, .02] | | |  |
| Mimicry × Behavior | 0.23 | | 2 | | 0.11 | | 0.11 | | .894 | | -.00 | | [.00, 1.00] | | |  |
| Error | 647.39 | | 647 | | 1.00 | |  | |  | |  | |  | | |  |
|  | |  | |  | |  | |  | |  | |  | |  | | |
| *Note*. LL and UL represent the lower-limit and upper-limit of the partial ω^2^ confidence interval, respectively. | | | | | | | | | | | | | | | | |

**Emotions.** In Study 1, we also measured participants’ emotions to control any potential effects of emotions on moral character judgments. Emotions were measure with 12 items: “anger”, “anxiety”, “contempt”, “disgust”, “frustration”, “guilt”, “shame”, “joy”, “happiness”, “arousal”, “interest”. Participants indicated to what extent they felt each emotion using a scale from 1 = *Not at all*to 7 = *A lot.*When we controlled for all emotions, moral information still moderated the effect of mimicry on moral character judgments, *F*(2, 635) = 3.64, *p* = .027.

**Study 2**

**Measures.**

**Trait perception** of the target person was measured as in Study 1. Participants indicated to what extent they agreed that the target person is warm (α = .89, *M* = 4.10, *SD* = 1.03), assertive (α = .69, *M* = 3.39, *SD* = 0.85) and competent (α = .88, *M* = 4.12, *SD* = 0.94) using a 7-point scale from 1 = *definitely not* to 7 = *definitely yes.*

**Results**

The **zero-order correlations** are presented in Table S10. The attitude was positively correlated with all other variables.

| Table S10 |  |  |  |  |
| --- | --- | --- | --- | --- |
|  |  |  |  |  |
| *Table of Correlations for Main Variables (Study 2)* | | |  |  |
|  |  |  |  |  |
| Variables | 1 | 2 | 3 | 4 |
| 1. Liking | - |  |  |  |
| 2. Morality | .68*** | - |  |  |
| 3. Warmth | .63*** | .80*** | - |  |
| 4. Assertiveness | .39*** | .34*** | .31*** | - |
| 5. Competence | .64*** | .64*** | .53*** | .60*** |
|  |  |  |  |  |
| *Note. *** p* < .001 | |  |  |  |

**Trait perception.** To test whether moral information would interact with liking on trait attributions different that moral ones, we performed a multivariate ANOVA with the preference manipulation (similar vs. dissimilar) and the information about the target’s past behavior (moral vs. control vs. immoral) as a between-subjects factors. This analysis confirmed that effects of liking for remaining traits were nonsignificant (see the full trait analysis for Study 2 including moral traits).

**Full trait analysis (Study 2)**

| Table S11 |  | |  | |  | |  | |  | |  | |  | |  | |  |  |
| --- | --- | --- | --- | --- | --- | --- | --- | --- | --- | --- | --- | --- | --- | --- | --- | --- | --- | --- |
|  | |  | |  | |  | |  | |  | |  | |  | |  | |  |
| *Means and standard deviations for the perception of moral traits as a function of a 2(preference) x 3(information) design (Study 2)* | | | | | | | | | | | | | | | | | | |
|  | |  | |  | |  | |  | |  | |  | |  | |  | |  |
|  | | Information | | | | | | | | | | | |  | |  | |  |
|  | | Moral | | | | Control | | | | Immoral | | | | Marginal | | | |  |
| Preference | | *M* | | *SD* | | *M* | | *SD* | | *M* | | *SD* | | *M* | | *SD* | |  |
| Similar | | 4.91 | | .95 | | 4.33 | | .83 | | 2.88 | | 1.10 | | 4.08 | | 1.28 | |  |
| Dissimilar | | 4.80 | | .92 | | 4.05 | | .81 | | 2.67 | | 1.02 | | 3.80 | | 1.28 | |  |
| Marginal | | 4.86 | | .94 | | 4.19 | | .83 | | 2.77 | | 1.06 | |  | |  | |  |
|  |  | |  | |  | |  | |  | |  | |  | |  | |  |  |
| *Note*. *M* and *SD* represents mean and standard deviation, respectively. | | | | | | | | | | | | |  | |  | |  |  |

| Table S12 |  | |  | |  | |  | |  | |  | |  | | |  |
| --- | --- | --- | --- | --- | --- | --- | --- | --- | --- | --- | --- | --- | --- | --- | --- | --- |
|  |  | |  | |  | |  | |  | |  | |  | | |  |
| *Fixed-Effects ANOVA results for the perception of moral traits (Study 2)* | | | | | | | | | | | | | | |  |  |
|  |  | |  | |  | |  | |  | |  | |  | | |  |
| Predictor | Sum of Squares | | *df* | | Mean Square | | *F* | | *p* | | ω^2^_p_ | | ω^2^_p_  95% CI [LL, UL] | | |  |
| (Intercept) | 9303.74 | | 1 | | 9303.74 | | 10419.25 | | < .001 | |  | |  | | |  |
| Information | 449.87 | | 2 | | 224.94 | | 251.91 | | < .001 | | .46 | | [.39, .51] | | |  |
| Preference | 5.65 | | 1 | | 5.65 | | 6.33 | | .012 | | .01 | | [.00, .03] | | |  |
| Information × Preference | .68 | | 2 | | .34 | | .38 | | .683 | | -.00 | | [.00, 1.00] | | |  |
| Error | 531.29 | | 595 | | .89 | |  | |  | |  | |  | | |  |
|  | |  | |  | |  | |  | |  | |  | |  | | |
| *Note*. LL and UL represent the lower-limit and upper-limit of the partial *ω^2^* confidence interval, respectively. | | | | | | | | | | | | | | | | |

| Table S13 |  | |  | |  | |  | |  | |  | |  | |  | |  |  |
| --- | --- | --- | --- | --- | --- | --- | --- | --- | --- | --- | --- | --- | --- | --- | --- | --- | --- | --- |
|  | |  | |  | |  | |  | |  | |  | |  | |  | |  |
| *Means and standard deviations for the perception of warmth traits as a function of a 2(preference) x 3(information) design (Study 2)* | | | | | | | | | | | | | | | | | | |
|  | |  | |  | |  | |  | |  | |  | |  | |  | |  |
|  | | Information | | | | | | | | | | | |  | |  | |  |
|  | | Moral | | | | Control | | | | Immoral | | | | Marginal | | | |  |
| Preference | | *M* | | *SD* | | *M* | | *SD* | | *M* | | *SD* | | *M* | | *SD* | |  |
| Similar | | 4.76 | | .83 | | 4.23 | | .84 | | 3.49 | | .97 | | 4.19 | | 1.02 | |  |
| Dissimilar | | 4.66 | | .82 | | 4.22 | | .79 | | 3.21 | | .86 | | 4.00 | | 1.02 | |  |
| Marginal | | 4.72 | | .83 | | 4.23 | | .81 | | 3.34 | | .92 | |  | |  | |  |
|  |  | |  | |  | |  | |  | |  | |  | |  | |  |  |
| *Note*. *M* and *SD* represents mean and standard deviation, respectively. | | | | | | | | | | | | |  | |  | |  |  |

| Table S14 | |  | |  | |  | |  | |  | |  | |  | | |  |
| --- | --- | --- | --- | --- | --- | --- | --- | --- | --- | --- | --- | --- | --- | --- | --- | --- | --- |
|  | |  | |  | |  | |  | |  | |  | |  | | |  |
| *Fixed-Effects ANOVA results for the perception of warmth traits (Study 2)* | | | | | | | | | | | | | | | |  |  |
|  |  | | |  | |  | |  | |  | |  | |  | | |  |
| Predictor | Sum of Squares | | | *df* | | Mean Square | | *F* | | *p* | | ω^2^_p_ | | ω^2^_p_  95% CI [LL, UL] | | |  |
| (Intercept) | 10046.50 | | | 1 | | 10046.50 | | 13869.23 | | < .001 | |  | |  | | |  |
| Information | 190.51 | | | 2 | | 95.26 | | 131.51 | | < .001 | | .30 | | [.24, .36] | | |  |
| Preference | 2.53 | | | 1 | | 2.52 | | 3.49 | | .062 | | .00 | | [.00, .02] | | |  |
| Information × Preference | 1.77 | | | 2 | | .89 | | 1.22 | | .295 | | .00 | | [.00, .01] | | |  |
| Error | 431.00 | | | 595 | | .72 | |  | |  | |  | |  | | |  |
|  | | |  | |  | |  | |  | |  | |  | |  | | |
| *Note*. LL and UL represent the lower-limit and upper-limit of the partial ω^2^ confidence interval, respectively. | | | | | | | | | | | | | | | | | |

| Table S15 |  | |  | |  | |  | |  | |  | |  | |  | |  |  |
| --- | --- | --- | --- | --- | --- | --- | --- | --- | --- | --- | --- | --- | --- | --- | --- | --- | --- | --- |
|  | |  | |  | |  | |  | |  | |  | |  | |  | |  |
| *Means and standard deviations for the perception of the assertiveness traits as a function of a 2(preference) x 3(information)design (Study 2)* | | | | | | | | | | | | | | | | | | |
|  | |  | |  | |  | |  | |  | |  | |  | |  | |  |
|  | | Information | | | | | | | | | | | |  | |  | |  |
|  | | Moral | | | | Control | | | | Immoral | | | | Marginal | | | |  |
| Preference | | *M* | | *SD* | | *M* | | *SD* | | *M* | | *SD* | | *M* | | *SD* | |  |
| Similar | | 3.70 | | .89 | | 3.35 | | .78 | | 3.40 | | .93 | | 3.49 | | .88 | |  |
| Dissimilar | | 3.44 | | .72 | | 3.17 | | .76 | | 3.29 | | .89 | | 3.30 | | .80 | |  |
| Marginal | | 3.58 | | .84 | | 3.26 | | .77 | | 3.34 | | .91 | |  | |  | |  |
|  |  | |  | |  | |  | |  | |  | |  | |  | |  |  |
| *Note*. *M* and *SD* represents mean and standard deviation, respectively. | | | | | | | | | | | | |  | |  | |  |  |

| Table S16 |  | |  | |  | |  | |  | |  | |  | | |  |
| --- | --- | --- | --- | --- | --- | --- | --- | --- | --- | --- | --- | --- | --- | --- | --- | --- |
|  |  | |  | |  | |  | |  | |  | |  | | |  |
| *Fixed-Effects ANOVA results for the perception of the assertiveness traits (Study 2)* | | | | | | | | | | | | | | |  |  |
|  |  | |  | |  | |  | |  | |  | |  | | |  |
| Predictor | Sum of Squares | | *df* | | Mean Square | | *F* | | *p* | | ω^2^_p_ | | ω^2^_p_  95% CI [LL, UL] | | |  |
| (Intercept) | 6897.98 | | 1 | | 6897.98 | | 9929.54 | | < .001 | |  | |  | | |  |
| Information | 10.21 | | 2 | | 5.11 | | 7.35 | | <.001 | | .02 | | [.00, .05] | | |  |
| Preference | 5.14 | | 1 | | 5.14 | | 7.40 | | .007 | | .01 | | [.00, .03] | | |  |
| Information × Preference | .60 | | 2 | | .30 | | .43 | | .650 | | -.00 | | [.00, 1.00] | | |  |
| Error | 413.34 | | 595 | | .70 | |  | |  | |  | |  | | |  |
|  | |  | |  | |  | |  | |  | |  | |  | | |
| *Note*. LL and UL represent the lower-limit and upper-limit of the partial ω^2^ confidence interval, respectively. | | | | | | | | | | | | | | | | |

| Table S17 |  | |  | |  | |  | |  | |  | |  | |  | |  |  |
| --- | --- | --- | --- | --- | --- | --- | --- | --- | --- | --- | --- | --- | --- | --- | --- | --- | --- | --- |
|  | |  | |  | |  | |  | |  | |  | |  | |  | |  |
| *Means and standard deviations for the perception of the competence traits as a function of a 2(preference) x 3(information) design (Study 2)* | | | | | | | | | | | | | | | | | | |
|  | |  | |  | |  | |  | |  | |  | |  | |  | |  |
|  | | Information | | | | | | | | | | | |  | |  | |  |
|  | | Moral | | | | Control | | | | Immoral | | | | Marginal | | | |  |
| Preference | | *M* | | *SD* | | *M* | | *SD* | | *M* | | *SD* | | *M* | | *SD* | |  |
| Similar | | 4.55 | | .91 | | 4.35 | | .79 | | 3.89 | | 1.04 | | 4.28 | | .96 | |  |
| Dissimilar | | 4.26 | | .77 | | 3.95 | | .91 | | 3.73 | | .92 | | 3.97 | | .90 | |  |
| Marginal | | 4.42 | | .86 | | 4.15 | | .88 | | 3.80 | | .98 | |  | |  | |  |
|  |  | |  | |  | |  | |  | |  | |  | |  | |  |  |
| *Note*. *M* and *SD* represents mean and standard deviation, respectively. | | | | | | | | | | | | |  | |  | |  |  |

| Table S18 | |  | |  | |  | |  | |  | |  | |  | | |  |
| --- | --- | --- | --- | --- | --- | --- | --- | --- | --- | --- | --- | --- | --- | --- | --- | --- | --- |
|  | |  | |  | |  | |  | |  | |  | |  | | |  |
| *Fixed-Effects ANOVA results for the perception of the competence traits (Study 2)* | | | | | | | | | | | | | | | |  |  |
|  |  | | |  | |  | |  | |  | |  | |  | | |  |
| Predictor | Sum of Squares | | | *df* | | Mean Square | | *F* | | *p* | | ω^2^_p_ | | ω^2^_p_  95% CI [LL, UL] | | |  |
| (Intercept) | 10171.32 | | | 1 | | 10171.32 | | 12628.70 | | < .001 | |  | |  | | |  |
| Information | 35.86 | | | 2 | | 17.93 | | 22.27 | | < .001 | | .07 | | [.03, .11] | | |  |
| Preference | 12.21 | | | 1 | | 12.21 | | 15.17 | | < .001 | | .02 | | [.01, .05] | | |  |
| Information × Preference | 1.47 | | | 2 | | .73 | | .91 | | .402 | | -.00 | | [.00, 1.00] | | |  |
| Error | 479.22 | | | 595 | | .81 | |  | |  | |  | |  | | |  |
|  | | |  | |  | |  | |  | |  | |  | |  | | |
| *Note*. LL and UL represent the lower-limit and upper-limit of the partial ω^2^ confidence interval, respectively. | | | | | | | | | | | | | | | | | |

**Study 3**

**Results**

| Table S19 | |  |  | |  | |  |
| --- | --- | --- | --- | --- | --- | --- | --- |
|  | |  |  | |  | |  |
| *Table of Correlations for Main Variables (Study 3)* | | | | |  | |  |
|  |  | | |  | |  | |
| Variables | 1 | | | 2 | | 3 | |
| 1. Liking mean | - | | |  | |  | |
| 2. Moral character judgement mean | .79** | | | - | |  | |
| 3. Attitude certianity mean | -.12* | | | -.12* | | - | |
| 4. Moral character judgement certianity mean | -.17** | | | -.22** | | .76** | |
|  |  | | |  | |  | |
| *Note. ** p* < .001; * *p* < .05 | | |  | |  | |  |

| Table S20 |  | |  | |  | |  | |  | |  | | |  | |  | |  |  |
| --- | --- | --- | --- | --- | --- | --- | --- | --- | --- | --- | --- | --- | --- | --- | --- | --- | --- | --- | --- |
|  | |  | |  | |  | |  | |  | |  | | |  | |  | |  |
| *Means and standard deviations for the mean of attitude certainty as a function of a 2 (attitude: similar vs. dissimilar) x 2 (moral information: first vs. second) design (Study 3)* | | | | | | | | | | | | | | | | | | | |
|  | |  | |  | |  | |  | |  | |  | | |  |  |  |  |  |
|  | | Moral information | | | | | | | | | | | | |  |  |  |  |  |
|  | | First | | | | Second | | | | Marginal | | |  |  |  |  |  |  |  |
| Attitude | | *M* | | *SD* | | *M* | | *SD* | | *M* | | *SD* |  |  |  |  |  |  |  |
| Similar | | 5.33 | | 1.18 | | 5.18 | | 1.17 | | 5.26 | | 1.18 |  |  |  |  |  |  |  |
| Dissimilar | | 5.18 | | 1.19 | | 4.75 | | 1.26 | | 4.96 | | 1.24 |  |  |  |  |  |  |  |
| Marginal | | 5.26 | | 1.19 | | 4.96 | | 1.23 | |  | |  |  |  |  |  |  |  |  |
|  |  | |  | |  | |  | |  | |  | | |  |  |  |  |  |  |
| *Note*. *M* and *SD* represents mean and standard deviation, respectively. | | | | | | | | | | | | | |  |  |  |  |  |  |

| Table S21 | |  | |  | | |  | |  | | | |  | | |  | | |  | | | | |
| --- | --- | --- | --- | --- | --- | --- | --- | --- | --- | --- | --- | --- | --- | --- | --- | --- | --- | --- | --- | --- | --- | --- | --- |
|  | |  | |  | | |  | |  | | | |  | | |  | | |  | | | | |
| *Fixed-Effects ANOVA results for the for the mean of attitude certainty (Study 3)* | | | | | | | | | | | | | | | | | | | | | | |  |
|  |  | | |  |  | | | | |  | |  | |  | | |  | | |  |  |  |  |
| Predictor | Sum of Squares | | | *df* | Mean Square | | | | | *F* | | *p* | | ω^2^_p_ | | | ω^2^_p_  95% CI [LL, UL] | | |  |  |  |  |
| (Intercept) | 20769.66 | | | 1 | 20769.66 | | | | | 7202.73 | | < .001 | |  | | |  | | |  |  |  |  |
| Information | 17.18 | | | 1 | 17.18 | | | | | 5.96 | | .015 | | .01 | | | [.00, .04] | | |  |  |  |  |
| Attitude | 17.26 | | | 1 | 17.26 | | | | | 5.99 | | .015 | | .01 | | | [.00, .04] | | |  |  |  |  |
| Information × Attitude | 3.79 | | | 1 | 3.79 | | | | | 1.31 | | .252 | | .00 | | | [.00, .02] | | |  |  |  |  |
| Error | 1136.13 | | | 394 | 2.88 | | | | |  | |  | |  | | |  | | |  |  |  |  |
|  | | |  | | |  | |  | | |  | | | |  | | |  | | |  |  |  |
| *Note*. LL and UL represent the lower-limit and upper-limit of the partial ω^2^ confidence interval, respectively. | | | | | | | | | | | | | | | | | | | | | |  |  |

| Table S22 | |  | | | |  | | |  | |  | | |  | | |  | | | |  | | | |  | |  |  |  |  |
| --- | --- | --- | --- | --- | --- | --- | --- | --- | --- | --- | --- | --- | --- | --- | --- | --- | --- | --- | --- | --- | --- | --- | --- | --- | --- | --- | --- | --- | --- | --- |
|  | | |  | | | |  | | |  | |  | | |  | | |  | | | |  | | | |  | |  |  |  |
| *Means and standard deviations for the mean of moral character judgement certainty as a function of a 2 (attitude: similar vs. dissimilar) x 2 (moral information: first vs. second) design (Study 3)* | | | | | | | | | | | | | | | | | | | | | | | | | | | | | | |
|  | | |  | | | |  | | |  | |  | | |  | | |  | | | |  |  |  |  |  |  |  |  |  |
|  | | | Moral information | | | | | | | | | | | | | | | | | | |  |  |  |  |  |  |  |  |  |
|  | | | First | | | | | | | Second | | | | | Marginal | | | | |  |  |  |  |  |  |  |  |  |  |  |
| Attitude | | | *M* | | | | *SD* | | | *M* | | *SD* | | | *M* | | | *SD* | |  |  |  |  |  |  |  |  |  |  |  |
| Similar | | | 5.33 | | | | 1.18 | | | 5.18 | | 1.17 | | | 5.26 | | | 1.18 | |  |  |  |  |  |  |  |  |  |  |  |
| Dissimilar | | | 5.18 | | | | 1.19 | | | 4.75 | | 1.26 | | | 4.96 | | | 1.24 | |  |  |  |  |  |  |  |  |  |  |  |
| Marginal | | | 5.26 | | | | 1.19 | | | 4.96 | | 1.23 | | |  | | |  | |  |  |  |  |  |  |  |  |  |  |  |
|  | |  | | | |  | | |  | |  | | |  | | |  | | | |  |  |  |  |  |  |  |  |  |  |
| *Note*. *M* and *SD* represents mean and standard deviation, respectively. | | | | | | | | | | | | | | | | | | | | |  |  |  |  |  |  |  |  |  |  |
| Table S23 |  | | |  | | | |  | | | | |  | | |  | | |  | | | | |  | | | | | |  |
|  |  | | |  | | | |  | | | | |  | | |  | | |  | | | | |  | | | | | |  |
| *Fixed-Effects ANOVA results for the for the mean of moral character jugdement certainty (Study 3)* | | | | | | | | | | | | | | | | | | | | | | | | | | | | |  |  |
|  |  | | | |  | | |  | | | | |  | | |  | | |  | | | |  | | | | | |  |  |
| Predictor | Sum of Squares | | | | *df* | | | Mean Square | | | | | *F* | | | *p* | | | ω^2^_p_ | | | | ω^2^_p_  95% CI [LL, UL] | | | | | |  |  |
| Certainity | 33.14 | | | | 1 | | | 33.14 | | | | | 31.08 | | | < .001 | | |  | | | |  | | | | | |  |  |
| Certainity x Moral information | 149.81 | | | | 1 | | | 149.81 | | | | | 140.53 | | | < .001 | | | .23 | | | | [.16, .31] | | | | | |  |  |
| Certainity x Attitude | 4.73 | | | | 1 | | | 4.73 | | | | | 4.44 | | | .036 | | | .01 | | | | [.00, .03] | | | | | |  |  |
| Certainity x Moral information x Attitude | 7.77 | | | | 1 | | | 7.77 | | | | | 7.29 | | | .007 | | | .01 | | | | [.00, .05] | | | | | |  |  |
| Error | 420.04 | | | | 394 | | | 1.07 | | | | |  | | |  | | |  | | | |  | | | | | |  |  |
| *Note*. LL and UL represent the lower-limit and upper-limit of the partial ω^2^ confidence interval, respectively. | | | | | | | | | | | | | | | | | | | | | | | | | | | | |  |  |

**Study materials**

**The assessment forms**. We pretested the assessment forms presented in Study 1 and Study 2 on 32 participants. Participants were randomly allocated in one of three conditions and presented with the assessment form in which the target’s supervisor mentioned the target’s moral behavior in the workplace (the moral condition), immoral behavior (the immoral condition) or information about the morality was omitted (the control condition). Afterward, participants judged how reliable and professional was the assessment, how competent was the target, and how moral was the target. Participants indicated the extent to which they agree with each of the statements using a scale from 1 = *I do not agree*to 7 = *I agree.*

There was no differences between the conditions in perception of reliability and professionalism of the assessment, *F*(2, 29) = .60, *p* = .553. Also, there was no differences between the conditions in perception of target’s competence, *F*(2, 29) = .02, *p* = .983. However, as we assumed there was the difference between the conditions in perception of target’s morality, *F*(2, 29) = 18.91, *p* < .001. Post-hoc comparisons revealed that in the immoral condition target was perceived as less moral (*M* = 2.84, *SD* = .67) than in the control condition (*M* = 4.54, *SD* = 1.07) and in the moral condition (*M* = 5.38, *SD* = 1.12, *ps* < .002).

**The assessment forms (Studies 1, 2 and 3)**

**The control condition**

**A descriptive evaluation of the employees’ work**

The employee began the work as a junior promotion sales specialist over eight months ago.

The employee has the right attitude towards the tasks but does not consistently deliver them correctly. The employee independently performs simple duties but requires supervision in more difficult ones. The employee usually meets the deadlines.

The employee can work in a team. Sometimes the employee burdens other team members with their tasks and duties. The employee is not always willing to help. Usually friendly but often shows impatience.

The employee is pretty good at work organisation. The employee can classify tasks and correctly determine the order of their execution if they are simple. Sometimes needs guidance in self-development. The employee acquires the minimum knowledge required for their position and improves their skills. The employee receives correct results on training, courses and other forms of professional development.

The employee tends to avoid independent action in crises. The employee works best under the guidance and successfully implements the proposed solutions. The employee does not always see areas for change. The employee uses motivation methods that are not always effective but are generally focused on achieving the goal. In general, the employee does not create conflict situations.

**The immoral condition**

**A descriptive evaluation of the employees’ work**

The employee began the work as a junior promotion sales specialist over eight months ago.

The employee has the right attitude towards the tasks but does not consistently deliver them correctly. The employee independently performs simple duties but requires supervision in more difficult ones. The employee usually meets the deadlines.

**The employee struggles with teamwork. The employee values their interests more than the interests of the team. The employee was found to use a fake sick leave, which allowed the employee to receive additional pay at the expense of the team’s salary.** The employee is not always willing to help. Usually friendly but often shows impatience.

The employee is pretty good at work organisation. The employee can classify tasks and correctly determine the order of their execution if they are simple. Sometimes needs guidance in self-development. The employee acquires the minimum knowledge required for their position and improves their skills. The employee receives correct results on training, courses and other forms of professional development.

The employee tends to avoid independent action in crises. The employee works best under the guidance and successfully implements the proposed solutions. The employee does not always see areas for change. **The employee does not set a good example and does not motivate other employees. The employee was found to alter the job sheet to hide being late in the workplace.** The employee uses motivation methods that are not always effective but are generally focused on achieving the goal. In general, the employee does not create conflict situations.

**The moral condition**

**A descriptive evaluation of the employees’ work**

The employee began the work as a junior promotion sales specialist over eight months ago.

The employee has the right attitude towards the tasks but does not consistently deliver them correctly. The employee independently performs simple duties but requires supervision in more difficult ones. The employee usually meets the deadlines.

**The employee appreciates the value of teamwork. The employee values interests of the team more than their intrests. The employee was found to share additional pay with younger team members who have offered help during the project execution.** The employee is not always willing to help. Usually friendly but often shows impatience.

The employee is pretty good at work organisation. The employee can classify tasks and correctly determine the order of their execution if they are simple. Sometimes needs guidance in self-development. The employee acquires the minimum knowledge required for their position and improves their skills. The employee receives correct results on training, courses and other forms of professional development.

The employee tends to avoid independent action in crises. The employee works best under the guidance and successfully implements the proposed solutions. The employee does not always see areas for change. **The employee sets a good example and motivates other employees.**

**The employee always admits to being late in the workplace and never altered the job sheet.** The employee uses motivation methods that are not always effective but are generally focused on achieving the goal. In general, the employee does not create conflict situations.

**Preference form (Study 2 and 3)**

Preference form was taken from previous studies by Sprecher (2019):

Which do you prefer? ___ Reality show ___ Sitcom

Which do you prefer? ___ Coffee ___ Tea

Which best describes you? ____Dreamer ____ Doer

Which do you prefer? ___ Radio ___ Spotify

Which best describes you? ___ Spender ___ Saver

Which do you prefer? ___ Frozen Yogurt ___ Ice cream

Which do you prefer? ___ Big party ___ Romantic dinner for 2

Do you? ___ Pay attention to details ____ Pay attention to the big picture

Which do you prefer? ___Mac ____ PC

Which best describes you? ___ Morning glory____ Night owl

Which one do you prefer to do? ___ Go with the flow ____ Stick to a routine

Which best describes you? ___ Worry wart ____ Worry free

Which do you prefer? ___ Museum of art ____ Museum of natural history

Which do you prefer? ___ Shop alone ____ Shop with others

Would you rather? ___ Watch TV ____ Read a book

Which one do you prefer? ____ Dark Chocolate ____ Milk Chocolate

Which best describes you? ____ Sloppy ____ Neat freak

**Reference**

Abele, A. E., Hauke, N., Peters, K., Louvet, E., Szymkow, A., & Duan, Y. (2016). Facets of the fundamental content dimensions: Agency with competence and assertiveness – communion with warmth and morality. *Frontiers in Psychology, 7*, 1810. https://doi.org/10.3389/fpsyg.2016.01810

Bocian, K., Baryla, W., Kulesza, W. M., Schnall, S., & Wojciszke, B. (2018). The mere liking effect: Attitudinal influences on judgments of moral character. *Journal Experimental Social Psychology,* *79*, 9-20. https://doi.org/10.1016/j.jesp.2018.06.007

Sprecher, S. (2019). Does (dis)similarity information about a new acquaintance lead to liking or repulsion? An experimental test of a classic social psychology issue. *Social Psychology Quarterly, 82,* 303–318. https://doi.org/10.1177/0190272519855954
